# Rapid weight loss in free ranging pygmy killer whales (*Feresa attenuata*) and the implications for anthropogenic disturbance of odontocetes

**DOI:** 10.1038/s41598-021-87514-2

**Published:** 2021-04-14

**Authors:** Jens J. Currie, Martin van Aswegen, Stephanie H. Stack, Kristi L. West, Fabien Vivier, Lars Bejder

**Affiliations:** 1 Pacific Whale Foundation, Wailuku, HI USA; 2 Marine Mammal Research Program, Hawaii Institute of Marine Biology, University of Hawaii at Manoa, Kaneohe, HI USA; 3 Hawaii Institute of Marine Biology, Kaneohe, HI USA; 4 Human Nutrition Food and Animal Sciences, College of Tropical Agriculture and Human Resources, Honolulu, HI USA; 5 Zoophysiology, Department of Biology, Aarhus University, Aarhus, Denmark; 6 Centre for Sustainable Aquatic Ecosystems, Harry Butler Institute, Murdoch University, Murdoch, WA Australia

**Keywords:** Animal behaviour, Conservation biology, Marine biology

## Abstract

Understanding the impacts of foraging disruptions to odontocete body condition is fundamental to quantifying biological effects of human disturbance and environmental changes on cetacean populations. Here, reductions in body volume of free-ranging pygmy killer whales (*Feresa attenuata*) were calculated using repeated measurements of the same individuals obtained through Unoccupied Aerial System (UAS)-photogrammetry during a prolonged disruption in foraging activity arising from a 21-day stranding event. Stranded individuals were used to verify UAS-derived volume and length estimates through 3D-imaging, water displacement, and post-mortem measurements. We show that (a) UAS estimates of length were within 1.5% of actual body length and UAS volume estimates were within 10–13% of actual volume, (b) foraging disruption resulted in a daily decrease of 2% of total body mass/day, and (c) pygmy killer whales can lose up to 27% of their total body weight within 17 days. These findings highlight the use of UAS as a promising new method to remotely monitor changes in body condition and animal health, which can be used to determine the potential effects of anthropogenic disturbance and environmental change on free-ranging odontocetes.

## Introduction

Anthropogenic disturbance can impact marine mammal behavior^1,2^ and vital rates^3–7^ which can lead to individual and population-level consequences^8^. In addition to altering resting and socializing behavior^2^, disturbances can also disrupt foraging activity^9^. Odontocetes have high metabolic rates, and as a result, individuals spend a considerable amount of time and energy on pursuing and capturing prey^10,11^. As such, disturbances that result in decreased foraging efficiency can result in reduced fitness of a population^12^. Quantifying the impacts of foraging disruptions on population health will lead to a better understanding of the biological significance of disturbance and how it affects vital rates^13,14^. Here we quantify the weight loss of free-ranging pygmy killer whales (*Feresa attenuata*) through repeated measurements of the same individuals during an extended disruption in foraging activity over 21 days. Working under the direction and authority of the National Oceanic and Atmospheric Administration, National Marine Fisheries Service (NOAA Fisheries), Marine Mammal Health and Stranding Response Program, we used Unoccupied Aerial System (UAS)-photogrammetry to obtain morphometric measurements to compare length and volume derived from the UAS-photogrammetry to post-mortem examination results of stranded individuals.

Reductions in body mass as a result of fasting have been documented in migrating and breeding baleen whales^14–16^ as well as captive harbor porpoises^17^. However, this metric is logistically difficult to obtain for free ranging odontocetes, yet represents a fundamental component of energetic-based approaches to estimating effects of disturbance, such as the Population Consequences of Disturbance (PCoD) model framework^18^.

The PCoD framework allows for the forecasting of population impacts by linking behavioral and physiological changes that occur in response to a disturbance^19^. To reduce forecasting uncertainty, detailed information on changes to behavior, physiology, health, and vital rates arising from disturbance are needed^6,19^. Disruptions to foraging behavior can have immediate impacts on individual physiology^17^, but have also been linked to long-term impacts on vital rates such as reduced reproduction and survival^7,20^. Knowledge of the physiological responses arising from altered environments is a key component when developing conservation strategies^21^ and contributes to the successful management of a species.

Changes in body condition have been calculated for free-ranging killer whales (*Orcinus orca*)^22^, however, minimal data exist on the changes in body condition of wild odontocetes. Given the difficulty of quantifying feeding requirements and the energetic needs for free-ranging odontocetes, most studies have largely relied on extrapolations^7^ with appropriate data for model validation lacking for many species^23^. The use of UASs to estimate morphometric characteristics is well established^24,25^ and has been used to quantify body conditions^26^ for a variety of marine mammals^27–29^. The initial application of UAS technology largely focused on photogrammetry of baleen whales^27^, but it is currently being used for a variety of research applications and species^30^, including odontocetes^22^. This work is an important step in expanding the use of UAS, particularly to quantify changes in body condition and individual health of odontocetes.

Numerous coastal populations of odontocetes experience disruptions to natural behavioral patterns arising from anthropogenic activities^2,5,13,31,32^. In Hawaii, 11 species of odontocetes are found within the coastal waters of the main Hawaiian Islands and rely on these waters as their primary foraging habitat^33^. With an estimated 80% of the 9.9 million people visiting Hawaii each year partaking in marine tourism^34^, the potential for interactions between humans and odontocetes in nearshore environments is high^35^. The lucrative nature of these interactions for some species^36^ is particularly problematic^37,38^, with high rates of disturbance observed in spinner dolphins (*Stenella longirostris*) targeted for close-up encounters^38,39^. This highlights the need to effectively predict and monitor potential consequences of disturbance to minimize the impacts of marine tourism on various odontocete species.

The cumulative impact of repeated non-lethal anthropogenic activities on wildlife populations is a growing concern. Here, we investigate the changes in pygmy killer whale body condition during a 21-day disruption in foraging activity resulting from a prolonged stranding event. We describe how these data can be used to inform on future work on non-lethal anthropogenic disturbances and contribute to effective management and conservation of small odontocetes.

## Results

### Timeline of stranding events and UAS-photogrammetry measurements

**Figure 1:**
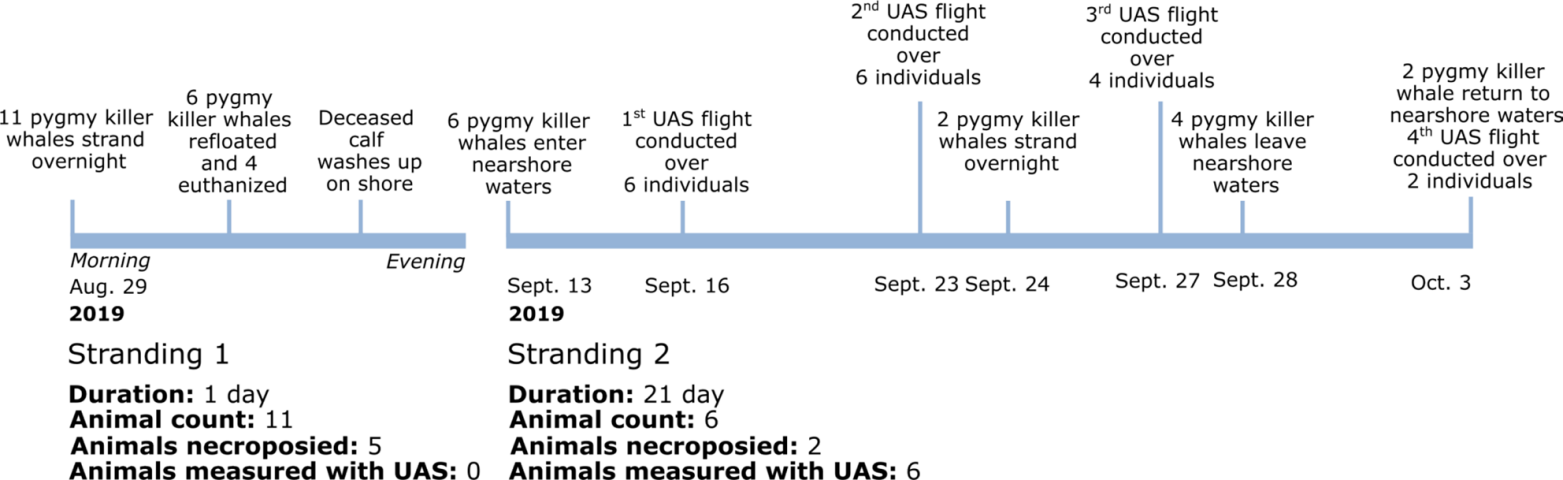
The timeline of two stranding events managed by NOAA Fisheries involving 17 pygmy killer whales *(Feresa attenuata)* that took place in the nearshore waters of Maʻalaea Bay, Maui between August and October 2019.

On August 29, 2019, NOAA Fisheries responded to an initial mass stranding (Stranding 1; Fig. 1) of 11 pygmy killer whales that had washed ashore in Maʻalaea Bay. Shortly after, on September 13, six pygmy killer whales appeared within 450 m of the shoreline (range 3–450 m) also in the Maʻalaea Bay, which ranges in depth from 3 to 30 m. The animals were outside their normal distribution, with those sighted in Hawaii usually found an average of 44 km offshore in water depths of ~ 1000 m^40,41^. The group was sighted in the nearshore shallow waters over 21 days, with consecutive daily sightings from September 13 to 28 (15 days; Fig. 1).

The body volumes of these six pygmy killer whales were estimated using UAS-photogrammetry over four sampling days (September 16, 23, 27 and October 3; Fig. 1). This occurred during a 21-day (September 13, 2019 to October 3, 2019) disruption to foraging activity (see results section on stomach contents from post-mortem examination below) in Maʻalaea Bay, Maui, Hawaii (Stranding 2; Supplementary Fig. S1). During the 21 day observation, two individuals (PKW2 and PKW3) stranded on shore on the morning of September 24. The remaining four individuals left the area in pairs on September 28 (PKW5 and PKW6) and October 4 (PKW1 and PKW4). While in the bay, daily monitoring of the animals revealed low energy behaviors (resting, milling, and slow movement), and tight group cohesion with ~ 2–5 m between individuals. The animals alternated between resting and slowly traveling at the surface.

### UAS-photogrammetry measurement accuracy

The mean variability in repeated length and volume calculations, determined using two independent photos per animal per day, across all individuals from UAS-derived measurements was ± 2.59 cm and ± 0.003 m^3^, respectively. UAS-derived lengths were within ~ 1.2% of actual lengths, while volumes were within 10.2–13.4% (Table 1). The animal’s extremities (dorsal fin, pectoral fin, and fluke) were not considered in the UAS-derived body volume estimate and accounted for ~ 2.3% of the total body volume (0.003 m^3^; Table 1).

**Table 1:** Pygmy killer whale length (m) and volume (m^3^) obtained from post-mortem water displacement and physical measurement compared to UAS-derived estimates that were collected on live animals at sea ~ 24 h prior to stranding on September 23, 2019.

Measurement parameters	PKW2	PKW3
**Length** (**m**)		
Post-mortem examination length measurements	2.35	2.32
UAS length	2.38	2.35
UAS measurement error (%)	1.27	1.29
**Volume** (**m**^**3**^)		
Post-mortem water displacement volume measurements	0.12	0.12
UAS 0–90% volume without extremities	0.13	0.13
UAS measurement error (%)	11.20	10.20
**Corrected × volume** (**m**^**3**^)		
Post-mortem water displacement volume measurements	0.12	0.12
Corrected UAS 0–90% volume with extremities	0.14	0.14
UAS measurement error (%)	13.43	12.40

Volume of fins obtained using 3D scans were added to UAS volume estimates to obtain corrected volume.

#### Correction of UAS-derived body volume estimates

A 3D-scan of a false killer whale *(Pseudorca crassidens)* was used to determine the difference in body volume from UAS-derived measurements as a result of excluding the animal's extremities (dorsal fin, pectoral fins, tail flukes) from volume estimates. For the purposes of this paper, it was assumed that the body proportions in false killer whales were similar to that of pygmy killer whales as the ratio of total length to extremities (i.e. pectoral fins, fluke and dorsal fin) differed by 0.5–3.0% between the two species^42,43^. The volume of the false killer whale’s extremities accounted for 1.59% of the UAS-derived body volume estimate. As such, the final UAS-derived volume estimates for PKW2 and PKW3 were increased by 1.59% of the estimated 90% body volume (Fig. 5B). Comparison of the volume recorded during post-mortem examinations with the corrected UAS estimates found that UAS data overestimated post-mortem examination volume by 10–13% (Table 1). Total length measurements derived from UAS data aligned closed with post-mortem measurements, differing by only 1.27% (Table 1).

### Changes in body volume over time

**Figure 2:**
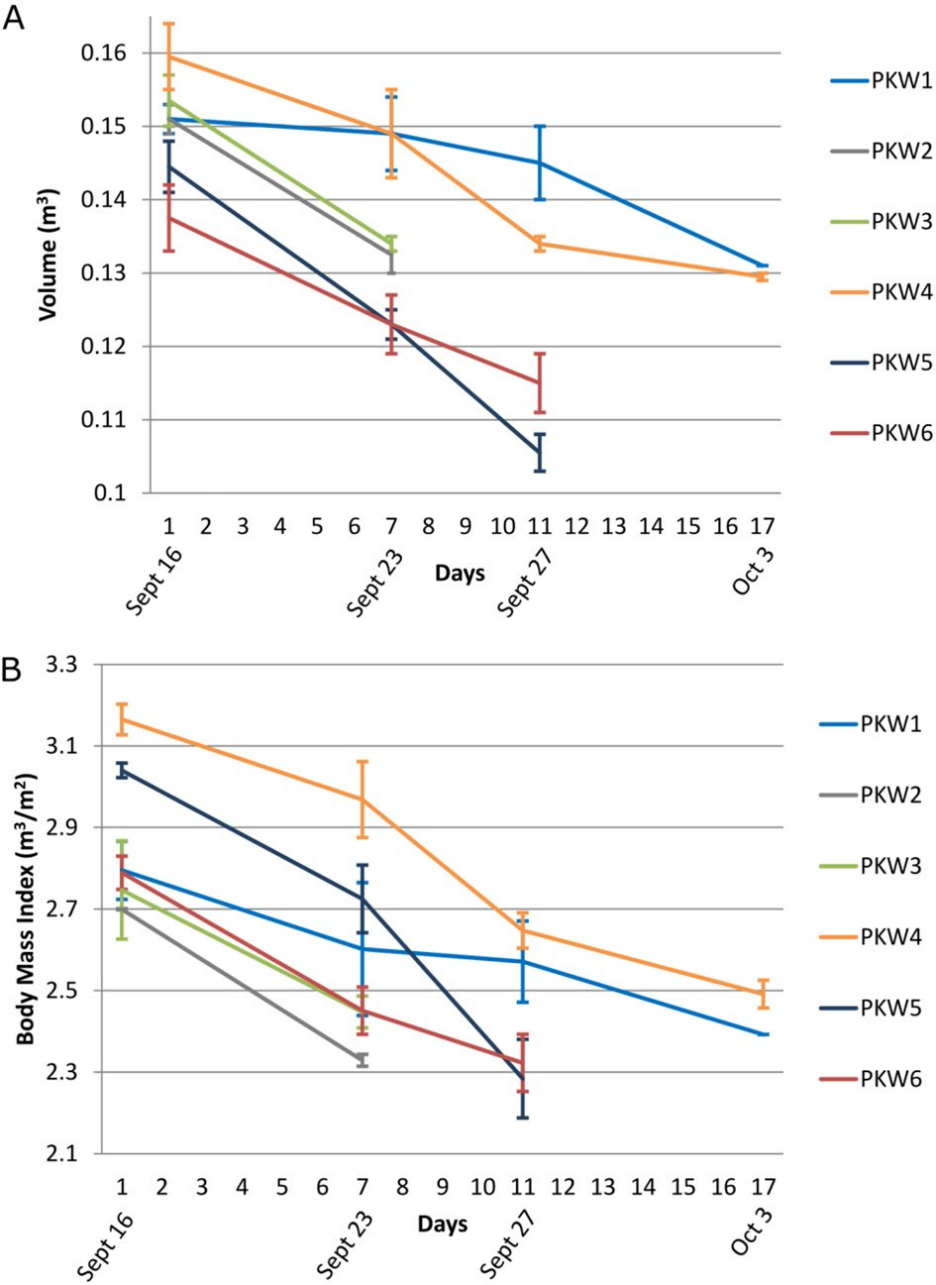
Pygmy killer whale (**A**) volume (m^3^) ± SD and (**B**) body mass index (BMI; m^3^/m^2^) ± SD as a function of observation days during a 21-day stranding event that took place from September 13 to October 3, 2019 in the nearshore waters of Maʻalaea Bay, Maui, where Day 1 corresponds to September 16, 2019. *Note*: UAS measurements were collected over the last 17 days of the stranding event.

All individuals experienced a decrease in body volume (Fig. 2) throughout the disruption in foraging and subsequent stranding event (Table 2). The smallest individual (PKW5) experienced the largest daily decrease in body volume (Table 2). The duration of observations varied from 7 to 17 days with mean daily body volume losses ranging from 0.80% (SD ± 0.56) to 2.58% (SD ± 0.45) of initial volume estimates (Table 2).

**Table 2:** Changes in body volume (m^3^) of six pygmy killer whales obtained using unoccupied aerial systems (UAS)-photogrammetry during a NOAA Fisheries response to a prolonged stranding event that took place from September 13 to October 3, 2019 in the nearshore waters of Maʻalaea Bay, Maui.

ID	Total length ± SD (m)	Total observation days (No. of UAS flights)	Average days between measurements (range)	Mean daily body volume change ± S.D	Total volume (m^3^) | mass loss (kg)
PKW1	2.36 ± 0.04	17 (4)	5.7 (4–7)	− 0.80% ± 0.56	0.020 | 23.23
PKW2	2.38 ± 0.02	7 (2)	7 (NA)	− 1.75% ± 0.00	0.019 | 20.63
PKW3	2.35 ± 0.02	7 (2)	7 (NA)	− 1.81% ± 0.00	0.020 | 23.55
PKW4	2.25 ± 0.03	17 (4)	5.7 (4–7)	− 1.25% ± 0.80	0.030 | 34.85
PKW5	2.15 ± 0.03	11 (3)	5.5 (4–7)	− 2.58% ± 0.45	0.039 | 45.30
PKW6	2.23 ± 0.02	11 (3)	5.5 (4–7)	− 1.48% ± 0.03	0.023 | 26.13

Loss in body volume ranged from 0.19 to 3.03% (SD ± 0.04) per day (Fig. 2), which is an estimated 0.33 to 5.09 kg with an assumed density of 1161.6 kg/m^3^ (see post-mortem examination methods) calculated using the average density of two stranded pygmy killer whales. It should be noted that the assumed density applied here is not likely representative of true animal’s density as varying stages of starvation will lead to varying rates of lipid and muscle metabolization, which will impact animal density. As such, it is important to consider these limitations during subsequent discussions of body mass calculations and subsequent energetic losses. The two individuals that stranded on shore (PKW2 and PKW3) lost an estimated 20.63 (SD ± 2.5) and 23.55 (SD ± 2.7) kilograms, respectively, within a one week period (Table 2; Fig. 2). Of all individuals, PKW5 experienced the greatest and most rapid weight loss of ~ 28.83 kg/week (SD ± 3.9) (Fig. 2).

### Change in body mass index over time

We calculated body mass index (BMI; volume/length^2^) to quantify potential changes to an animal’s condition between sightings. All individuals exhibited reductions in energy stores over the sampling period, with average BMIs of 2.87 (range 2.70–3.16) and 2.37 (range 2.28–2.49) observed between first and last sightings (Fig. 2B). The BMI scores decreased by an average of 20.86% between first and last sightings, with a minimum observed reduction of 12.22% and maximum of 33.40%. The greatest overall percent change in body mass index (33.40%) was observed in PKW5, which also experienced the largest reduction in body volume (0.039 m^3^; Table 2) and subsequent mass (45.30 kg).

### Findings from post-mortem examinations

Once deceased, individuals PKW2 and PKW3 were recovered by NOAA Fisheries and post-mortem examinations were conducted at the University of Hawaii Health and Stranding Laboratory. Stomach content analyses suggested abnormal foraging efforts that are outside of the expected diet of pygmy killer whales, which is thought to be primarily cephalopods^44^. Individual PKW2 had remains of small unidentifiable fish and both marine and terrestrial plant material. Individual PKW3 also had remains of small unidentifiable fish, as well as ten leafy green structures and an undigested Moorish idol (*Zanclus cornutus*) in the esophagus. Five other pygmy killer whales that were necropsied following the August 29^th^ mass stranding event did not have any prey remains in their stomach with the exception of one individual (presence of a few fish otoliths and a nematode). The cause of the 2019 prolonged pygmy killer whale mass stranding events remains unknown.

**Figure 3:**
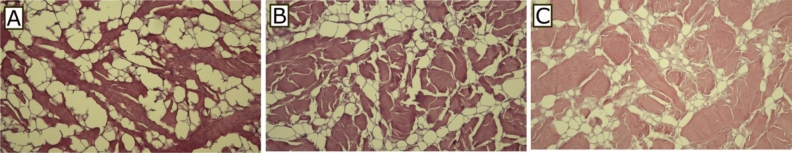
Adipocyte images (white is lipid—filled adipocyte; red is intervacuolar space) from blubber samples obtained during post-mortem examination of a non-fasting (**A**) and two fasting (**B**,**C**) male pygmy killer whales that stranded on August 29 (**A**) and September 24, 2019 (**B**,**C**) in Maʻalaea Bay, Maui.

Adipocyte index values^45^ used as a proxy for animal health were calculated for PKW2 and PKW3 and indicated low energy reserves (Table 3). The adipocyte index values for PKW2 and PKW3 were compared with that of a sub-adult pygmy killer whale that stranded during August 29, 2019 (PKW7; Fig. 1). During the post-mortem examination, the August 29 sub-adult male pygmy killer whale had an empty stomach, but this individual is assumed to represent an animal that had not experienced a prolonged foraging disruption as it was not previously observed in nearshore waters prior to the initial mass stranding event. When the adipocyte index values were compared between the August 29 non-fasting sub-adult pygmy killer whale and the fasting adults PKW2 and PKW3, a percent difference of 84% for PKW2 and 105% for PKW3 were observed (Fig. 3; Table 3).

**Table 3:** Summary of post-mortem examination results of three pygmy killer whales comparing a non-fasting individual (PKW7) that stranded on August 29, 2019 to likely-fasting individuals, PKW2 and PKW3, that stranded on September 23, 2019 after 11 continuous days of observation and retrieval by NOAA Fisheries in the nearshore waters of Maʻalaea Bay, Maui.

Individual	PKW2	PKW3	PKW7
Sex	Male	Male	Male
Length (m)	2.35	2.32	2.13
Weight (kg)	131.50	146.50	109.30
Density (kg/m^3^)	1115.35	1207.75	*NA*
Adipocyte index	1.81	2.37	0.74

### Volume loss in relation to estimated daily energetic requirements

The loss of body volume, determined here using UAS-based photogrammetry and arising from disruption to foraging, was found to have implications for animal health and survival (Table 3; Fig. 3). Based on the weights of PKW2 and PKW3 and the resting metabolic rate presented in^46^, the daily energetic requirements of PKW2 and PKW3 at the time of stranding were estimated to be 3550.5 and 3955.5 kcal/day, respectively. It is important to note that these estimates assume the resting metabolic rates taken from bottlenose dolphins^46^ are comparable for pygmy killer whales in this study and this limitation needs to be considered when interpreting daily energetic calculations. With initial body weights calculated to be 152.1 kg and 170.1 kg for PKW2 and for PKW3, respectively, the disruption in foraging resulted in a daily average weight loss of 2.9–3.4 kg, or ~ 2% of the total body mass per day.

## Discussion

This study used UAS-photogrammetry to quantify volume and weight loss in six free-ranging pygmy killer whales to determine implications for foraging disruptions. We compared UAS-derived estimates of volume and length to values determined from post-mortem examinations to refine UAS measurement accuracy. The rapid weight loss observed during the disruption to foraging highlights the rapid rate at which energy reserves can become depleted in small odontocetes when feeding is stopped or interrupted. Although previous recommendations of monitoring behavioral changes from disturbance over extended periods^47^ are still warranted, this study highlights that even short-term monitoring (< 7 days) of foraging disruption can result in measurable changes in body condition. The effectiveness of UAS-photogrammetry as a tool to detect volume changes and accurately measure body length highlights the use of this approach in monitoring the health and body condition of free-ranging odontocetes. The observed overestimation of body volume from true volume arises from the use of a circular cross-section body shape for volume estimates, which does not represent the true shape of pygmy killer whales^28,48^. Additional work has shown cetaceans to be most circular in the mid region, with variations from this shape near the anterior and posterior ends of the body^28,48^. Despite this overestimation in true body volume the 0–90% UAS-derived body volumes were measured with a high-degree of accuracy with significantly different UAS 0–90% body volumes detected within 4 days.

Here, we show that free-ranging pygmy killer whales lost ~ 2% of their body mass per day when they ceased normal foraging. The diet of pygmy killer whales in Hawaiian waters is unknown but cephalopods have been described as the dominant dietary item from stranded pygmy killer whales off of the Gulf of California, Mexico^44^ and were absent among the stomach contents of PKW2 and PKW3. The rate of weight loss as a result of feeding disruption has previously been studied for captive harbor porpoises (*Phocoena phocoena*) where animals lost 1.3–2.4 kg/day or approximately 4% of their total body mass^17^. As pygmy killer whales are nearly four times the mass of harbor porpoises, these data suggest that odontocetes with larger body sizes may be more resilient to disruptions in feeding and potential weight loss over time as seen in other cetacean species^49,50^. This is further supported by the results presented here, which found the smallest individual (PKW5) had the largest and fasted decline in body volume. The ability to detect changes in body volume/mass using UAS for small odontocetes will depend on the severity of the disturbance (i.e., foraging disrupted, foraging delayed, etc.) and further research is needed in this area. Bioenergetic models developed for sperm whales (*Physeter macrocephalus*) found individual resilience to foraging disruptions is primarily a function of size (i.e. reserve capacity)^12^. Indeed, larger baleen whales may be more resilient to disturbance that results in foraging disruptions as some species, such as southern right whales (*Eubalaena australis*), regularly lose ~ 25% of their body volume as part of their life history strategy^16^. Despite this, disturbances during these prolonged fasting events for large baleen whales can limit their ability to replenish energy reserves and/or complete various life cycle functions.

Responses to disturbance that result in reduced or ceased foraging activity are more commonly reported for odontocetes^51^. However, this may represent a lack of long-term data on mysticete disturbance. Regardless, the higher metabolic rates observed in odontocetes^10^, could result in a more immediate and noticeable impact on animal health and energy. Reduced foraging efficiency can extend the time it takes to reach terminal starvation^12^. This may have been observed here as abnormal prey items were found in two of the stranded animals 12 days after a disruption to foraging was observed. Even frequent disruptions of foraging at low levels can be fatal^12^ and highlights the need for incorporating the potential energetic consequences arising from occasional or frequent disruption in foraging into management plans.

Activity state plays a crucial role in regulating the loss of body mass, with metabolic rates in active adult bottlenose dolphins 3–6 times that of resting animals^46^. Although behavioral budgets were not quantified, the observation of primarily low energy behaviors, such as milling and resting, in these animals throughout the 21-day stranding event likely slowed weight loss. This highlights the importance of ensuring animals that have experienced a disturbance or displacement from their preferred habitat do not experience further stress, which may result in unnecessary energy expenditure.

**Figure 4:**
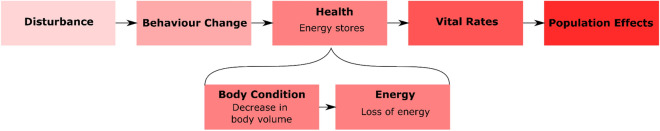
Modified overview of the Population Consequences of Disturbances (PCoD) framework with insert showing where UAS-photogrammetry can be used to quantify changes in body volume and make inferences about energy loss and animal health. Figure has been modified from^4^.

Since its development in 2005, The Population Consequences of Disturbance (PCoD) model^6,18,19^ has gone through various iterations to better define mechanistic links between disturbances and consequences^47^ with an emphasis on linking behavior change to body condition^13^. Here, we provide information on the rate of volume loss (and hence body condition) as well as known volumes and densities in free-ranging pygmy killer whales, which can be used to quantify the mechanistic link between energy loss and disturbance (foraging disruption) in similar sized odontocetes (Fig. 4). Individual health through the calculation of an adipocyte index^45^ conducted for fasting (PKW2, PKW3) and non-fasting (PKW7) individuals aligns with expectations that non-fasting individuals would have larger blubber lipid stores, as demonstrated by the lower adipocyte index. The effectiveness of this metric in odontocetes supports its use for monitoring cetacean population lipid stores and highlights the potential for broader species applications. Further, evidence of marked adipocyte atrophy, which is indicative of dramatic lipid mobilization, aligns with observed prolonged fasting to the point of indiscriminate foraging behavior and ingestion of plant material.

With increasing awareness on the implication of non-lethal effects of human disturbance and environmental changes^52^ on cetaceans^6,14^, mechanisms to understand the relationship between disturbance that results in disruptions in foraging, and animal health are increasingly important. Future applications of UAS-photogrammetry to detect changes in body volume for odontocetes should consider comparisons of impacted and non-impacted populations to investigate long-term implication of disturbance on body condition. In this study, anthropogenic disturbance leading to the observed foraging disruption was not documented and it is unknown in this case what led to the prolonged stranding event. The ability to detect small changes in body condition has been demonstrated using various techniques^26^ and the ability to detect them over a short period of time, as presented here, can be applied to determine the potential impacts of other human-caused interactions. These could include commercial fisheries, chemical pollution, military sonar, and vessel traffic that bring odontocetes into direct conflict with humans and can lead to reduced fitness, disturbance, and disruptions in foraging efficiency^9,32,32,51,53^. Research findings from this study will help validate model extrapolations used in the PCoD framework and determine the type and amount of sampling required to effectively model energy loss from foraging disruption that could negatively affect free-ranging marine mammals. Future research should focus on determining the applicability of generalizing weight loss reported here across similar sized odontocetes.

To date, there have been few studies utilizing UAS to accurately measure the body volume of free-ranging odontocetes and detect changes in body condition over time. A clear understanding between body condition, energy stores, foraging disruption and the potential cause of disturbance is important for quantifying the effects of anthropogenic activities on vital rates and population dynamics^54,55^. The animal densities, volumes, and weights presented here, in conjunction with detectable changes in body condition over short time periods (4–6 days) arising from a disruption in foraging, are key components in the PCoD modeling framework. These data are now available to provide a mechanistic link between energy loss and the type of foraging disruption that could result from anthropogenic disturbance to be used in the development of effective management and conservation strategies for odontocetes.

## Methods

### Data collection approvals

The data collected during the 21-day prolonged pygmy killer whale stranding event was part of the overall event response efforts coordinated by NOAA Fisheries under research and enhancement permit number 18786-03.

The NOAA Fisheries research permit number 21321 issued to Pacific Whale Foundation approved UAS flights and research methodologies. Flights were performed in compliance with Federal Aviation Administration guidelines with UAS pilot licensed with Part 107 authorization. The research activities were performed in accordance with the guidelines and regulations outlined above. Informed consent has been obtained for publication of images containing identifying features of individuals.

### Unoccupied aerial systems data collection and processing

High-resolution (3840 × 2160 pixels) aerial videos and subsequent still-images were obtained using a UAS. A DJI Inspire 2 quadcopter with a Zenmuse X5S gimbal camera and 25 mm f1.8 lens was flown, launching from either a research vessel or land, at an altitude of 15 m above surfacing pygmy killer whales. UAS altitude used in subsequent image analysis was recorded using a custom-built altimeter system based on^56^, which measured the height of the UAS above water on a one second interval. Still-images (8 megapixels) were extracted from the video and selected when animals were extending their body, with both the tip of the rostrum and fluke notch visible at the water surface, with minimal roll, pitch and arching^16^.

### UAS-photogrammetry measurement of body volume and length

**Figure 5:**
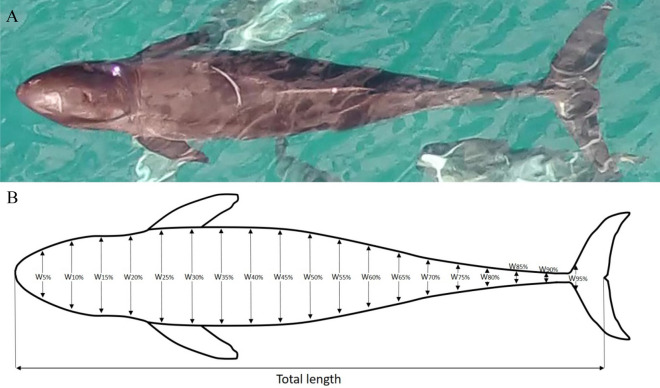
Example of an (**A**) aerial photograph taken from an unoccupied aerial system showing required body orientation at the surface to be used in analysis and (**B**) diagram noting the positions used to calculated total length and the 0–90% body volume. The diagram was made using Microsoft PowerPoint 2010 <https://www.microsoft.com/en-us/microsoft-365/powerpoint>.

Total body length (tip of rostrum to the fluke notch; TL) and body widths (at 5% increments all along the body axis) were measured for each individual (Fig. 5). Multiple scarring and dorsal fin characteristics unique to each animal were used to identify and track individuals from the air. Dorsal characteristics (scars, nicks, notches) captured by the UAS were cross-referenced with vessel-derived photo-identification images, allowing for repeat aerial measurements of individuals over time. To maximize sample size and estimate potential measurement errors, two independent still-images were extracted for each individual pygmy killer whale present on a given day. All measurements were made using custom-written MATLAB software designed to account for camera lens distortion^56^. Images were scaled using both known focal length (25 mm) and UAS altitude data recorded using custom-built altimeter. Image quality was quantified using the grading system developed in previous work^16^, which included assigning numerical scores 1 (good), 2 (medium) and 3 (poor) to six attributes including camera focus, body roll, body pitch, body arch, body length measurability and body width measurability. One attribute, body straightness, was not included, as our measurement software successfully accounts for curved body contours. Any image attributes with a score of 3 were removed from analysis. Using methods previously described in the literature^16^, body volume calculations were made using 19 body-width measurements along the body axis between 0 and 90% of body length (Fig. 5). Assuming a circular cross-sectional body shape, the volume for each 5% segment was calculated and summed to estimate body volume.

### Testing UAS measurement consistency and accuracy

**Figure 6:**
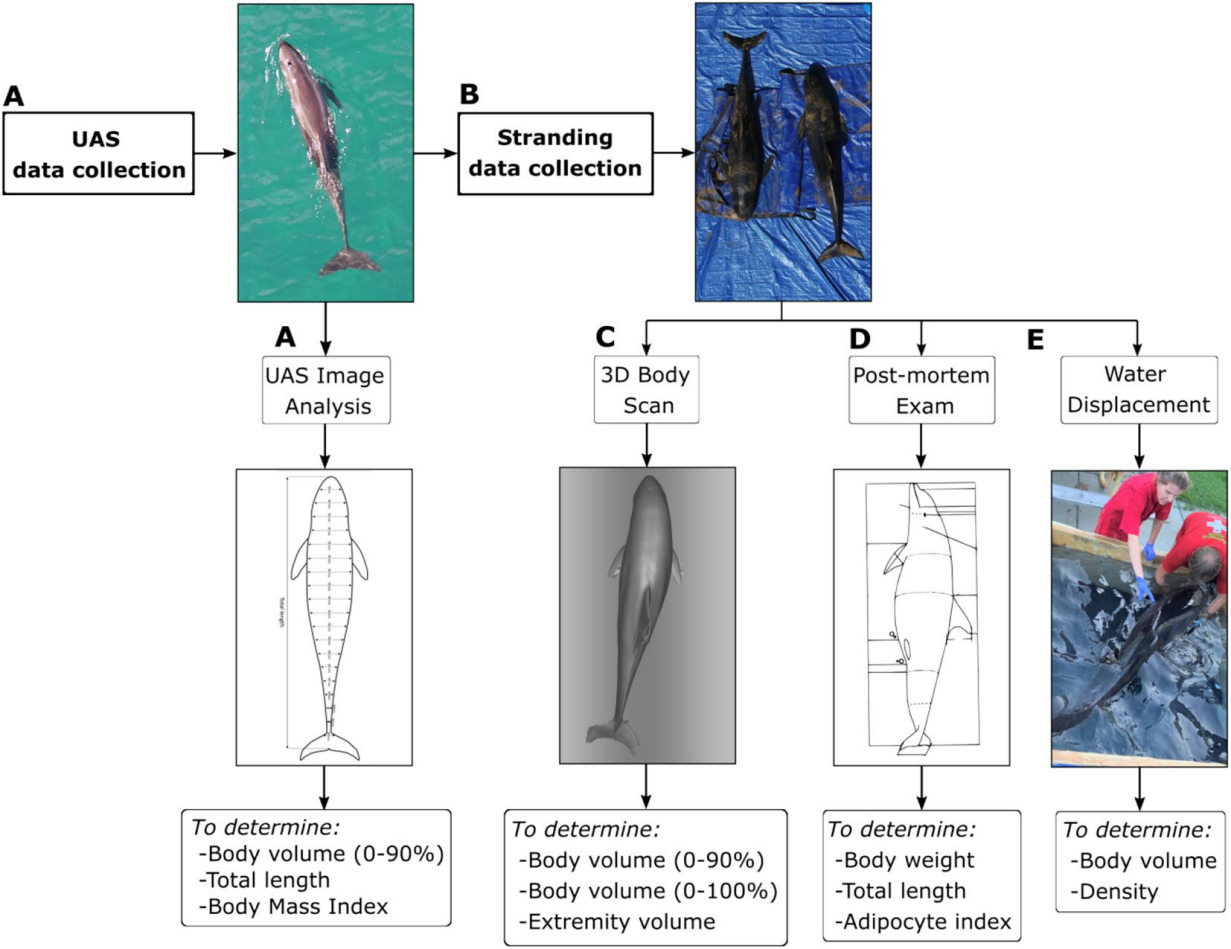
Workflow to collect UAS-derived measurements of body volume and length (**A**) and determine UAS measurement consistency and accuracy through stranding data (**B**) by conducting post-mortem examinations and 3D scans (**C**–**E**). *3D scan was conducted on a false killer whale (Pseudorca crassidens).

To determine the consistency and accuracy of UAS data, repeated body volume and length measurements of free swimming individuals collected from UAS were compared to body volumes and length determined from post-mortem examination and water displacement. The exclusion of extremities (dorsal, pectoral, flukes) from UAS measurements were corrected using of a 3D scanned false killer whale to determine extremity volumes (Fig. 6).

The mean variability in UAS-derived length and volume measurements was calculated by completing two independent measurements from different images of each individual per day, with the average of the two measurements used in subsequent analysis. PKW2 and PKW3 stranded 19 h after being measured while free-swimming with the UAS and were necropsied ~ 11 h after stranding. These two individuals form the basis for our comparison of UAS-derived length and volume estimates with post-mortem examination measurements. To ensure an accurate comparison between the water displacement volume measurements (included extremities volumes) with the UAS-derived volume estimates (excluded extremities volumes) a volumetric correction factor was calculated. This was done using a 3D-scan of a similar sized odontocete, a sub-adult male false killer whale. The 3D-image (Supplementary Fig. S2) was created by scanning a false killer whale that died in a fishery interaction and was frozen for subsequent necropsy using a 3D-scanner (Leo, Artec3D) with a precision of a volume precision of 0.03% over 100 cm. For full 3D-reconstruction, the animal was placed on a table to allow for unobstructed access to the lateral and dorsal parts of the body. Once these parts were scanned, the animal was rotated on a lateral side to allow for ventral scanning.

Once all parts of the body were scanned, the corresponding scans were processed and merged in Artec Studio 14 Professional (Artec3D) to recreate the full shape of the animal. The scan alignment consisted of designating three to five corresponding points on two separate scans to allow for the alignment based on the points selected (i.e., the main points used consisted of the blowhole, the eyes, the tips and base of the fins, the notch in the flukes and any significant scar or marks on the body). Subsequent scans were then aligned to the previously merged scans which allowed for a full 3D-reconstruction of the animal. Secondly, any hole in the 3D-reconstructed shape was filled to create a watertight object (required to calculate the volume of an object). The volumes of the extremities (dorsal fin, pectoral fins, and flukes) and body volume, to match UAS-derived measurements, were calculated separately and the percent of body volume the extremities accounted for was determined.

### Calculation of body mass index (BMI)

Body mass index (BMI) is a morphometric index commonly applied to make quantitative inferences about the condition of an animal^57,58^. In other odontocete species, higher BMI values indicate an animal in relatively better condition, while the inverse represents a less-optimal body condition^57^. Often calculated by dividing mass with length, this index can be misleading when comparing individuals of different sizes, given mass often scales with body length^59^. Here, we utilized an adapted BMI equation presented in^59^ to quantify relative changes in the condition of animals of different body lengths. While the revised equation also divides mass with length, the latter is squared, resulting in a condition index that is more independent of body size relative to mass/length alone^59^:

$$BMI = \left( {\frac{V}{{TL^{2} }}} \right)*100$$

where *V* is the body volume estimate and *TL* is the total length estimate obtained for an animal in an image. The mean BMI for each individual on a given day was calculated by averaging the two BMI estimates available for each individual animal per observation.

### Post-mortem examinations: data collection and processing

On September 24th, 2019, two of the six pygmy killer whales (PKW2 and PKW3) live-stranded on Sugar Beach, Maʻalaea Bay, Maui (Supplementary Fig. S1) at first light and were pushed back to sea by members of the public. NOAA Fisheries responded and PKW2 and PKW3 subsequently re-stranded a short time later and were humanely euthanized. The carcasses were recovered by NOAA Fisheries, chilled, and transported to Oahu for data collection, post-mortem examinations and sample analyses at the University of Hawaii Health and Stranding Laboratory. Prior to necropsy, water displacement was conducted with each carcass using a fiberglass vessel that measured 167 cm × 150 cm with a maximum height of 72 cm. The vessel was filled approximately 75% full with water and the fill line marked on the inside of the water displacement vessel. Each carcass was then placed gently in the vessel and submerged underwater and the subsequent fill line was marked. The difference in fill lines were measured using digital calipers and the dimensions of the vessel was used to calculate the total water volume displaced by each carcass. Morphometric data and total body weight were obtained from each pygmy killer whale prior to necropsy which focused on stomach content examination for prey identification, external and internal gross examination, internal organ weights and extensive formalin fixed and frozen tissue collections for histopathology and disease screening. The two pygmy killer whales were determined to be mature adult males based on testes size and histological examination that confirmed evidence of spermatogenesis.

For adipocyte area and index analysis, each blubber sample was collected from a standardized location anterior to the dorsal fin in PKW2 and PKW3 as well as in a sub-adult male pygmy killer whale (PKW7) that stranded on August 29th, 2019 as part of the first mass stranding event. Approximately 100 mg of blubber tissue was sampled and frozen at necropsy at − 20 °C within 13 h post-mortem. Blubber was later thawed and samples were fixed in 10% neutrally buffered formalin, paraffin embedded and sectioned at 4–5 μm intervals using a rotary microtome and stained with haematoxylin and eoxin (HE). Images were taken using a Jentopix Gryphax Arktur digital camera mounted on Olympus BX41 microscope at 10 × magnification and image processing was conducted in the software project Fiji (Image J) using the Adiposoft software plug in. Blubber adipocyte area was calculated by measuring a minimum of 100 adipocytes from three images of the middle blubber layer and averaging the areas^45^. Blubber adipocyte index is a measure of the ratio of connective tissue to adipocyte volume. Similar to blubber adipocyte area calculations, adipocyte index was obtained from three images of the middle blubber layer and then averaged for each individual. Adipocyte index has previously been suggested as an important measure of adiposity (the higher the adipocyte index, the lower the adiposity) for humpback whales and an important tool in the monitoring of their population health^43^.

Densities were determined using the animal’s volume calculated through water displacement, and weight measured using a scale. Similar densities were assumed across individuals (PKW1, PKW4, PKW5, and PKW6), based on the average determined from (PKW2 and PKW3), which were of comparable size. The average density was used to convert UAS-derived changes in body volume (m^3^), to changes in body mass (kg). This approach uses the best available data, but it is important to note that is assumes that densities of the stranded pygmy killer whales are comparable to the non-stranded whales and that the body densities of the non-stranded whales remained constant throughout the UAS measurements. However, different stages of starvation will result in varying levels of blubber volumes and subsequent densities and, as such, is a source of variability in subsequent body mass calculations and energetic expenditures.

### Calculating resting metabolic rate

The daily energetic requirements for pygmy killer whales are unknown and for the purposes of this paper, we used data from resting free-ranging bottlenose dolphins (*Tursiops truncatus*) sampled in the wild^46^, through a capture-release program. A resting metabolic rate from wild dolphins was considered most appropriate given the majority of observed pygmy killer whale activity consisted of resting behavior. The resting metabolic rate was assumed to be 27 kcal/kg/day, which was determined for a bottlenose dolphin of comparable weight (150 kg) to the observed pygmy killer whales^46^. Individual energetic requirements (kcal/day) were calculated by multiplying the above resting metabolic rate by mass of the animal. It should be noted that calculations of energetic requirements assumed that the resting metabolic rate of bottlenose dolphins was comparable to the pygmy killer whales measured in this study. This in conjunction with the limitations around calculation of animal density needs to be considered when interpreting energetic requirements and weight loss.

## Supplementary Information

Supplementary Information
