# Supplementary material for: Rapid weight loss in free ranging pygmy killer whales (Feresa attenuata) and the implications for anthropogenic disturbance of odontocetes
Source: Sci Rep. 2021 Apr 14;11:8181. doi: 10.1038/s41598-021-87514-2 (PMC8046785; doi:10.1038/s41598-021-87514-2)
Supplement: Supplementary file 1 — Supplementary Information [file 41598_2021_87514_MOESM1_ESM.docx]

Rapid weight loss in free ranging pygmy killer whales (*Feresa attenuata*) and the implications for anthropogenic disturbance of odontocetes

Jens J. Currie^1*^, Martin van Aswegen^2^, Stephanie H. Stack^1^, Kristi L. West^,3,4^, Fabien Vivier^2^, Lars Bejder^2,5,6^

^1^Pacific Whale Foundation, Wailuku, HI, USA

^2^ Marine Mammal Research Program, Hawaii Institute of Marine Biology, University of Hawaii at Manoa, HI, USA

^3^ Hawaii Institute of Marine Biology, HI, USA

^4^ Human Nutrition Food and Animal Sciences, College of Tropical Agriculture and Human Resources, HI, USA

^5^Zoophysiology, Department of Biology, Aarhus University, Denmark

^6^Centre for Sustainable Aquatic Ecosystems, Harry Butler Institute, Murdoch University, Western Australia


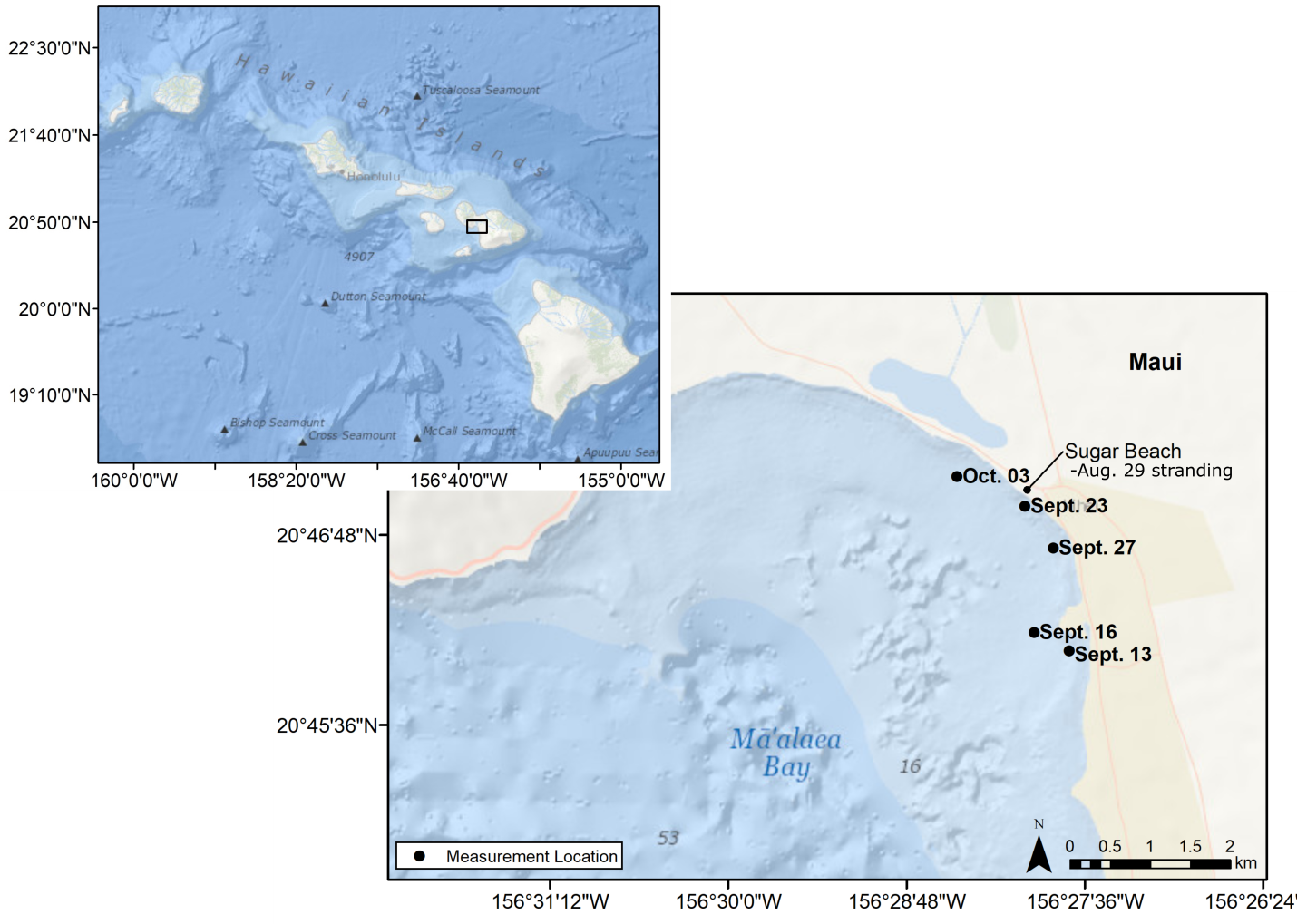


Supplementary Figure 1. Map showing location and date of UAS measurements of pygmy killer whales taken between September 13 and October 3 2019 in Maʻalaea Bay, Maui, as well as the Sugar Beach stranding location. The map was created using ArcGIS version 10 software^1^ with the ocean base map sourced from: Esri, GEBCO, NOAA, National Geographic, DeLorme, HERE, Geonames <http://services.arcgisonline.com/arcgis/rest/services/Ocean/World_Ocean_Base/MapServer>

1.ESRI 2012. ArcGIS Desktop: Release 10. Redlands, CA: Environmental Systems Research Institute.


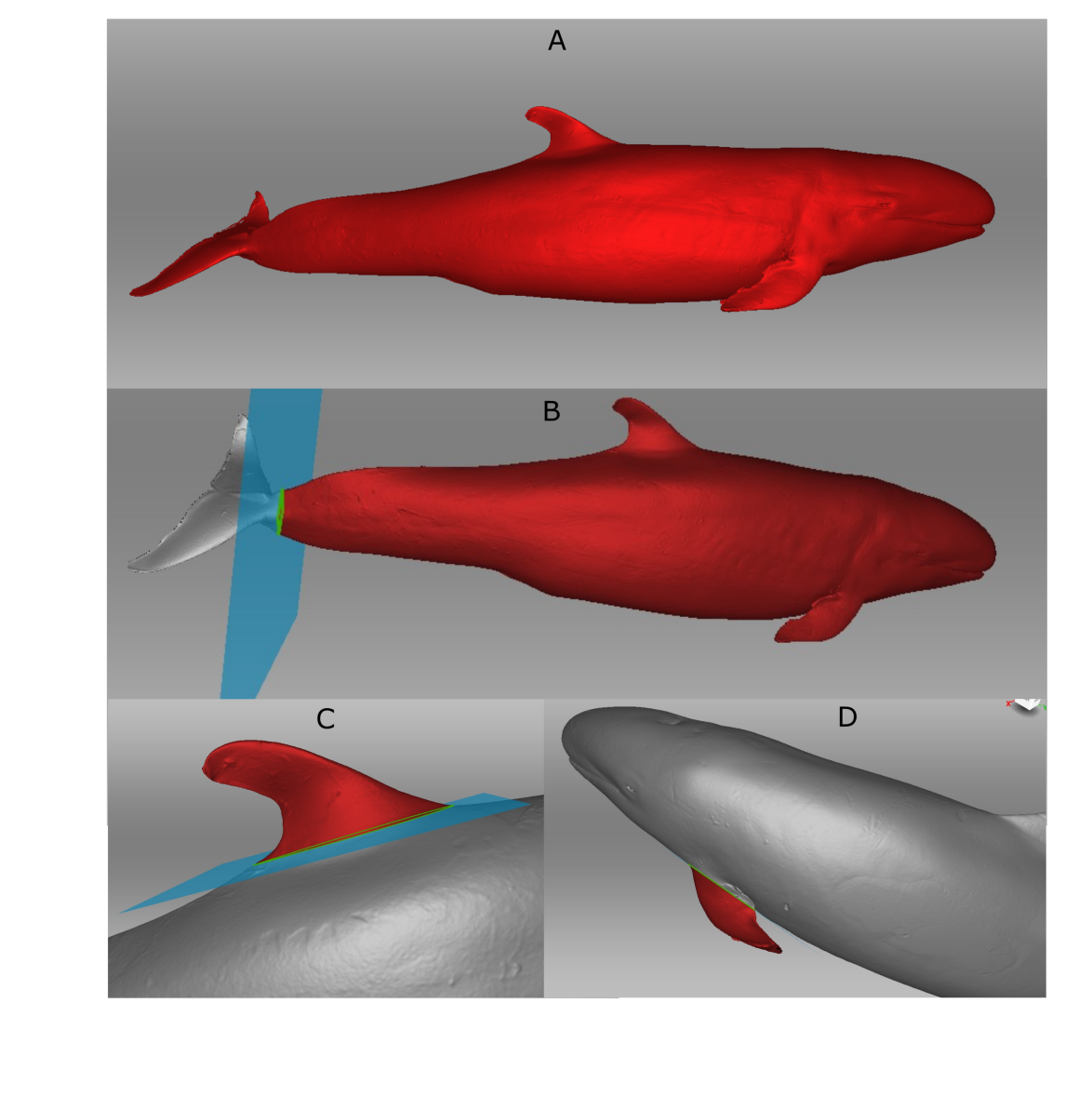


Supplementary Figure 2. 3-D scans of a deceased false killer whale (*Pseudorca crassidens*) used to calculate (A) total body volume (m^3^), with examples of (B) 0-90% body volume, (C) dorsal fin volume, and (D) left pectoral fin volume shown. The images were created using Artec Studio 14 Professional (Artec3D) < https://www.artec3d.com/3d-software/artec-studio>
